# Successful treatment of grade III traumatic pancreatic injury with non-operative management: a case report

**DOI:** 10.1093/jscr/rjae722

**Published:** 2024-11-25

**Authors:** Kazuki Matsushita, Atsushi Urakami, Munenori Takaoka, Katsunori Ishii, Tomohiro Tanikawa, Hirofumi Kawamoto, Tomoki Yamatsuji

**Affiliations:** Department of General Surgery, Kawasaki Medical School General Medical Center, 2-6-1 Nakasange, Kita-ku, Okayama 700-8505, Japan; Department of General Surgery, Kawasaki Medical School General Medical Center, 2-6-1 Nakasange, Kita-ku, Okayama 700-8505, Japan; Department of General Surgery, Kawasaki Medical School General Medical Center, 2-6-1 Nakasange, Kita-ku, Okayama 700-8505, Japan; Department of General Internal Medicine 2, Kawasaki Medical School General Medical Center, 2-6-1 Nakasange, Kita-ku, Okayama 700-8505, Japan; Department of General Internal Medicine 2, Kawasaki Medical School General Medical Center, 2-6-1 Nakasange, Kita-ku, Okayama 700-8505, Japan; Department of General Internal Medicine 2, Kawasaki Medical School General Medical Center, 2-6-1 Nakasange, Kita-ku, Okayama 700-8505, Japan; Department of General Surgery, Kawasaki Medical School General Medical Center, 2-6-1 Nakasange, Kita-ku, Okayama 700-8505, Japan

**Keywords:** traumatic pancreatic injury, endoscopic drainage, pancreatic duct drainage, pseudocyst drainage, pancreatic pseudocyst, traffic trauma

## Abstract

According to the American Association for the Surgery of Trauma, distal pancreatectomy or pancreatic duct drainage is recommended for grade III traumatic pancreatic injuries. We report a case of traumatic pancreatic injury involving the main pancreatic duct in which this method failed to drain fluid from the area distal to the injury site. A 19-year-old woman presented with a bruised upper left abdomen after a bicycle fall. Computed tomography revealed a linear area of poor contrast in the pancreatic body, leading to the diagnosis of grade III pancreatic injury. Endoscopic retrograde pancreatography revealed damage to the pancreatic duct, prompting endoscopic pancreatic stent placement. We added abdominal cavity drainage, peritoneal lavage, and endoscopic ultrasound-guided transgastric pseudocyst drainage. In the patient with pancreatic duct injury, drainage distal to the injury site was unattainable with a pancreatic duct stent; therefore, alternative drainage sites were utilized, thereby obviating the need for surgery.

## Introduction

Pancreatic trauma is rarer than injuries affecting other solid organs of the abdomen, accounting for 0.2%–0.3% of trauma cases [1, 2]. Based on the classification of the American Association for the Surgery of Trauma, distal pancreatectomy or pancreatic duct drainage is recommended for grade III traumatic pancreatic injuries [3].

Herein, we present a case of grade III traumatic pancreatic injury with main pancreatic duct injury in which drainage distal to the injury site was unattainable with a pancreatic duct stent. We treated the patient using an endoscopic pancreatic stent (EPS), endoscopic ultrasound (EUS)-guided transgastric pseudocyst drainage, and peritoneal lavage with abdominal cavity drainage, which prevented the need for surgical intervention.

## Case report

**Figure 1:**
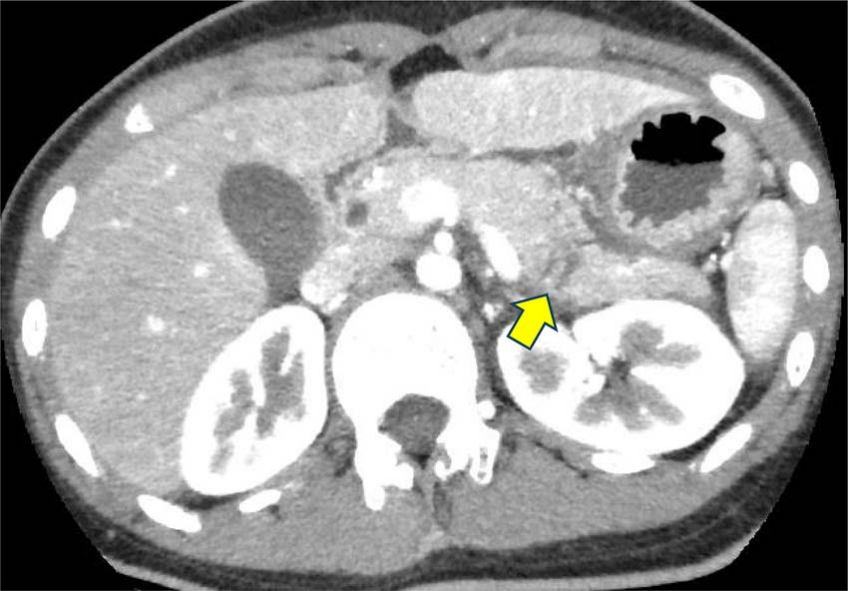
Contrast-enhanced CT on admission. Linear areas with poor contrast in the pancreatic body and increased peripancreatic fatty tissue density were observed.

**Figure 2:**
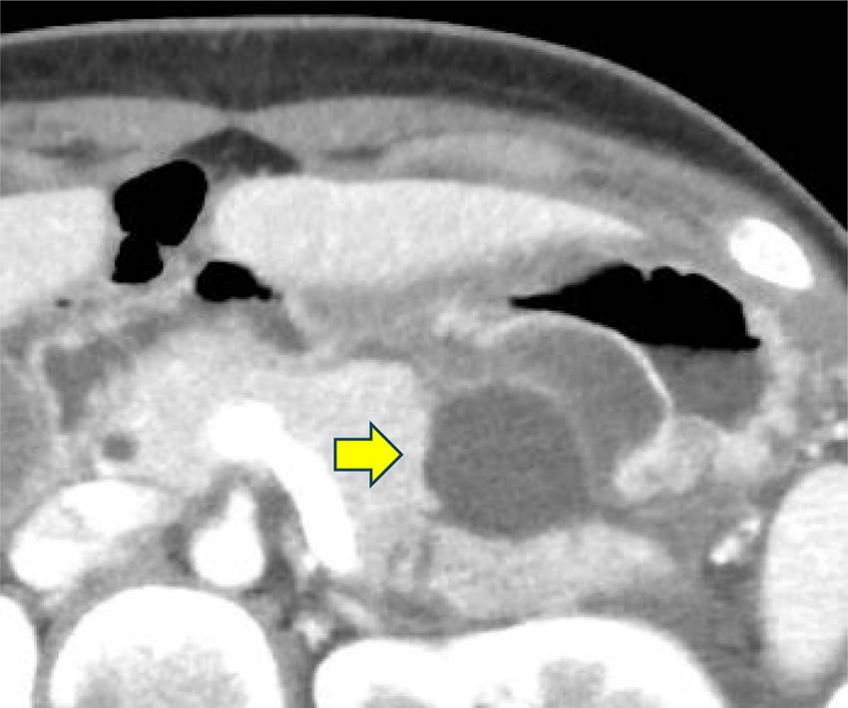
Contrast-enhanced CT of the abdomen on the fourth day. Fluid accumulation (pancreatic pseudocyst) was observed, primarily located on the ventral pancreatic body and extending toward the vicinity of the spleen, along with edematous thickening of the adjacent gastric posterior wall.

A 19-year-old woman who had fallen from a bicycle and bruised her upper left abdomen with handlebars presented to our emergency department 18 h after the injury with persistent abdominal pain. She was admitted with a diagnosis of pancreatic injury, as evidenced by elevated pancreatic amylase (P-Amy) levels (779 IU/L) and contrast-enhanced computed tomography (CT) showing a linear area with poor contrast in the pancreatic body (Fig. 1). Physical examination revealed a flat abdomen with mild tenderness in the upper left quadrant. Biochemical blood tests revealed elevated inflammatory markers; white blood cell count 10 950/μl, and C-reactive protein 0.58 mg/dl. On the fourth day, white blood cell count elevated to 21 940/μl and C-reactive protein elevated to 32.7 mg/dl. Contrast-enhanced CT revealed fluid accumulation, indicating pancreatic pseudocyst, primarily in the ventral pancreatic body and extending toward the spleen, along with edematous thickening of the adjacent stomach wall (Fig. 2).

**Figure 3:**
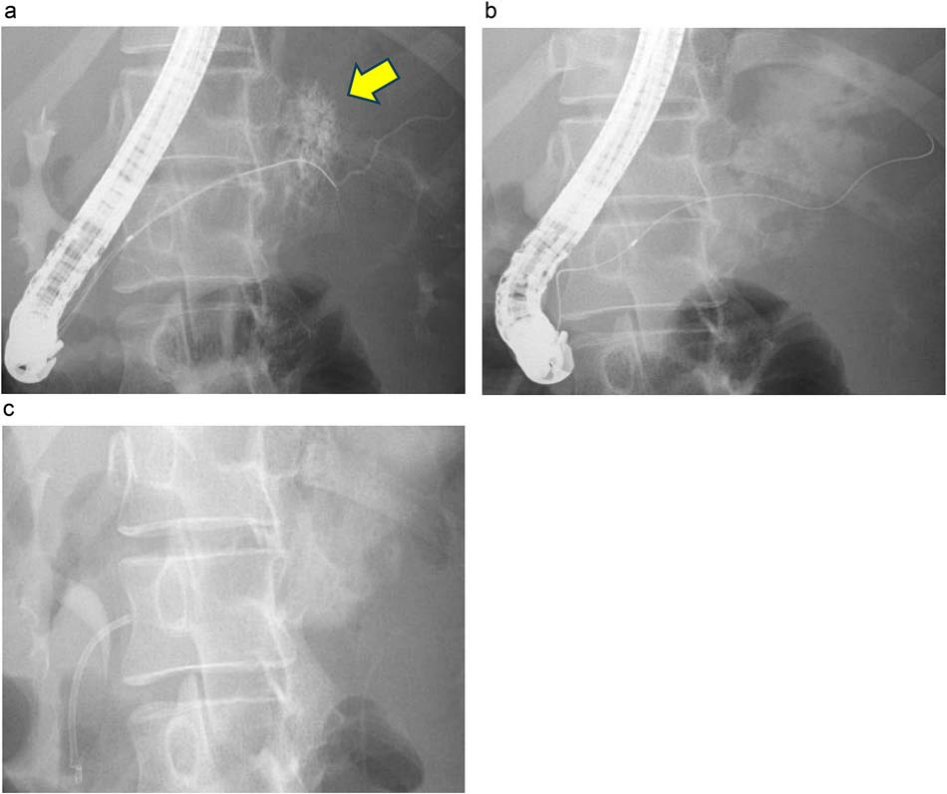
ERP and EPS on the fourth day. (a) Leakage of the contrast medium was observed in the pancreatic body, leading to the diagnosis of grade III pancreatic injury. (b) The cannula had difficulty passing through the injury site; however, the guidewire was observed to have passed beyond the pancreatic duct injury site at the pancreatic tail. (c) A 5 Fr-5 cm EPS was placed in the region of the pancreatic head.

Endoscopic retrograde pancreatography (ERP) was performed, and contrast leakage was observed in the pancreatic body, leading to the diagnosis of grade III pancreatic injury (Fig. 3a). Although the guidewire was successfully passed beyond the injury site to the caudal pancreatic duct, cannula passage proved challenging (Fig. 3b). Consequently, a 5 Fr EPS was placed in the region of the pancreatic head (Fig. 3c).

**Figure 4:**
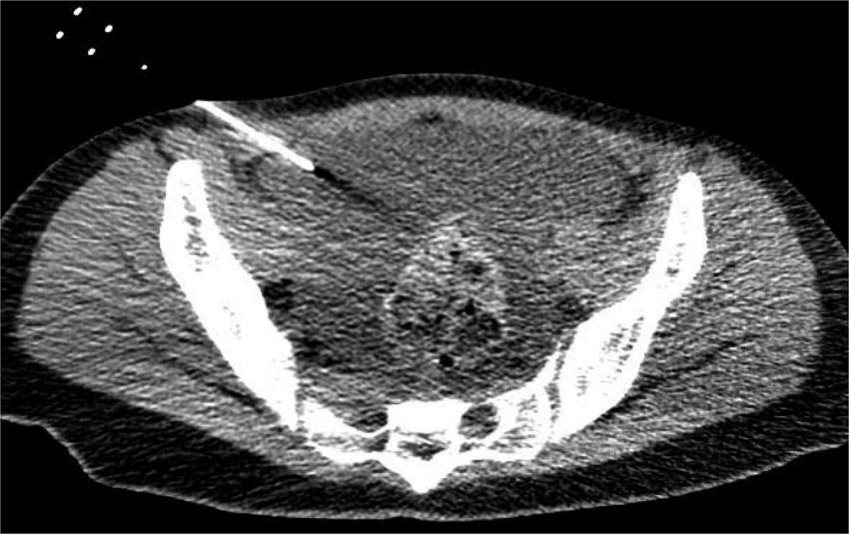
CT of the pelvic region on the fourth day. An increase in ascitic fluid was observed. CT-guided abdominal drainage was performed, and a 10 Fr drainage tube was placed.

**Figure 5:**
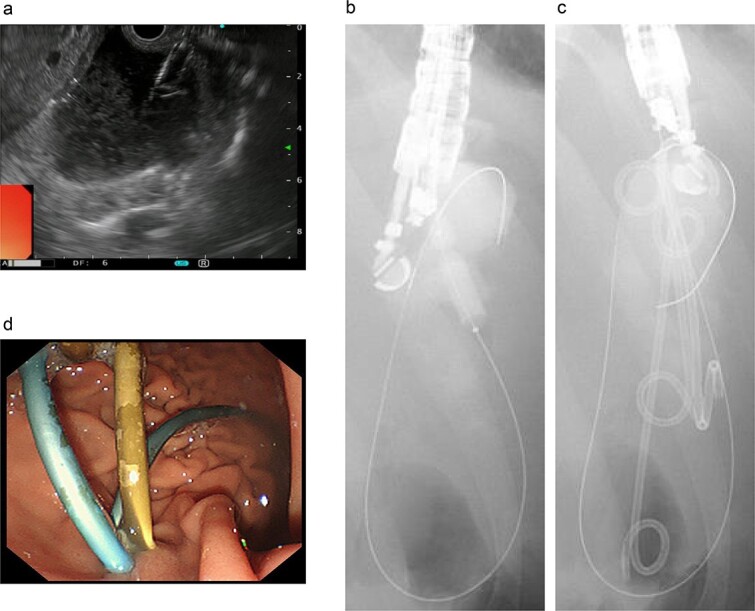
Endoscopic ultrasound-guided transgastric pseudocyst drainage. (a) Endoscopic ultrasound-guided puncture of the pseudocyst. (b) The posterior wall of the stomach was punctured, and the puncture hole was dilated with a balloon. (c) Placement of three tube stents through the puncture hole. (d) Placement of three tube stents of 7 Fr-7 cm and 7 Fr-4 cm × 2.

**Figure 6:**
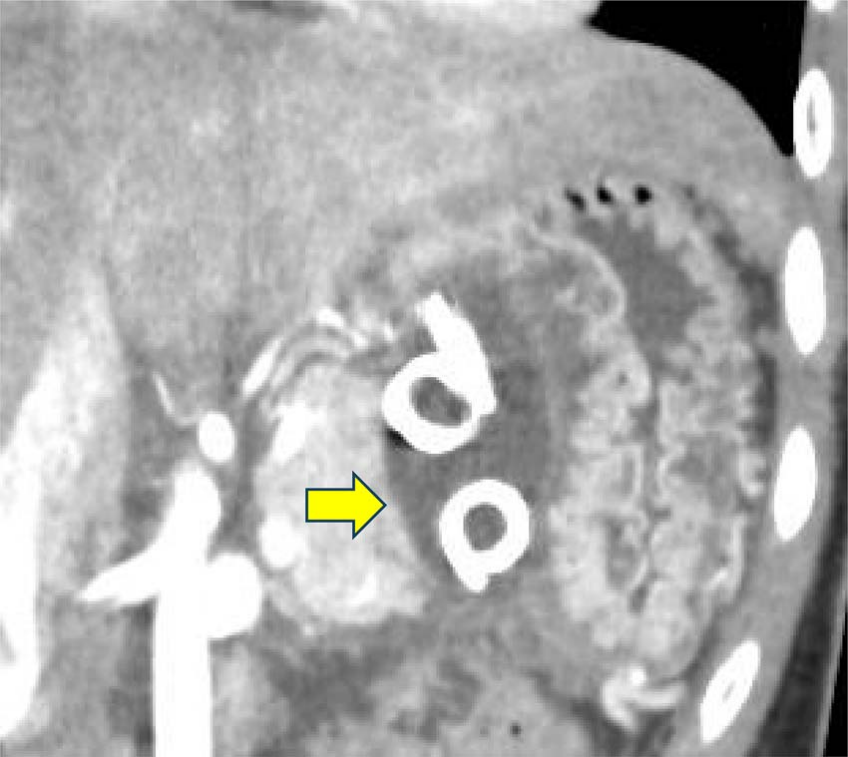
Contrast-enhanced CT on the 12th day. The pseudocyst in the omental bursa had shrunk. The arrow indicates the pseudocyst.

**Figure 7:**
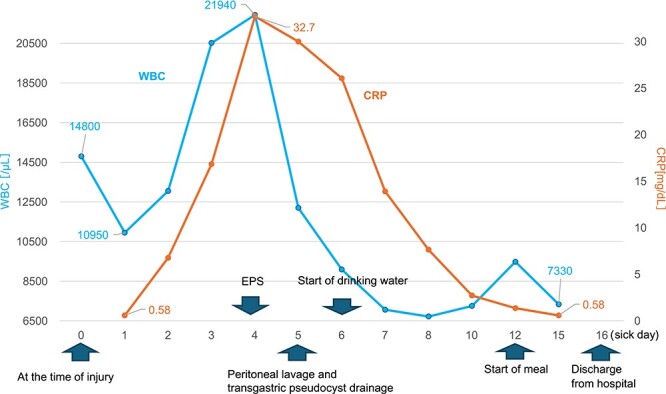
Changes in the white blood cell count and C-reactive protein (CRP) levels over time. The graph shows the changes in white blood cell (WBC) count and CRP levels over time from admission to discharge.

On the fifth day, the ascitic fluid increased in volume, and an open distal pancreatectomy was considered. However, her condition was stable. CT-guided abdominal drainage was performed, and a 10 Fr drainage tube was inserted for peritoneal lavage (Fig. 4). EUS-guided transgastric drainage of the pseudocyst in the omental bursa was performed. The pseudocyst was punctured; a balloon was dilated through the posterior wall of the stomach (Fig. 5a–c), and three tube stents of 7 Fr were implanted (Fig. 5d). The Amy and P-Amy levels in ascites were 9429 and 8877 IU/L, respectively; then, peritoneal lavage was performed daily. On the 12th day, contrast-enhanced CT revealed reduced pseudocyst size (Fig. 6). The patient was discharged on the 16th day (Fig. 7). Three years later, the patient is living her daily life without any complications.

## Discussion

The key determinant in the treatment of pancreatic trauma is the presence or absence of damage to the main pancreatic duct. Non-operative management (NOM) is often feasible if there is no main pancreatic duct injury. Conversely, when there is a main pancreatic duct injury, aggressive surgical interventions, such as pancreatectomy or main duct repair, are necessary [4, 5]. Nevertheless, a previous case of grade III traumatic pancreatic injury was initially managed using pancreatic duct drainage, followed by transgastric drainage under EUS for a pancreatic pseudocyst, successfully managing the pancreatic injury with NOM [6, 7]. The efficacy of transgastrointestinal drainage under EUS has been increasingly recognized in cases in which other approaches are challenging.

ERP has demonstrated efficacy as an initial treatment modality [8] given its ability to accurately diagnose the location and extent of pancreatic duct injury and facilitate the insertion of a pancreatic duct stent. A critical consideration in pancreatic stent insertion is whether drainage beyond the injury to the distal pancreatic duct is feasible. If drainage is only achievable proximal to the injury site, surgical intervention is preferable because the volume of pancreatic fluid leakage is substantial and may lead to potential complications [9, 10]. Conversely, if drainage can be performed distal to the injury, NOM is likely to yield favorable outcomes [7]. Nonetheless, vigilant and prompt monitoring for the potential development of pancreatic pseudocysts after the acute phase remains crucial.

Owing to the position of the pancreas on the posterior wall of the omental bursa, damage to the pancreatic surface membrane and parenchyma from pancreatic duct injury can lead to the leakage of pancreatic fluid into the omental bursa, resulting in pseudocyst formation [11]. Consequently, if endoscopic transgastric drainage can be performed from the posterior wall of the stomach, which serves as the anterior wall of the omental bursa, favorable drainage outcomes can be anticipated. Additionally, because it constitutes an internal fistula, this approach can preserve the quality of life of the patient [12].

In this case, it was difficult to place a stent beyond the site of the pancreatic duct rupture; therefore, a pancreatic duct stent was placed in the region of the pancreatic head. Subsequently, persistent leakage of pancreatic juice from the injured main pancreatic duct resulted in the formation of a pseudocyst in the omental bursa. The pancreatic fluid leaked from the omental bursa into the abdominal cavity via the foramen of Winslow, resulting in increased pancreatic ascitic fluid. Intraperitoneal drainage, repeated peritoneal lavages, and EUS-guided transgastric drainage were performed. The symptoms improved rapidly after drainage, and the levels of both inflammatory markers and pancreatic enzymes improved, suggesting the efficacy of all three drainage sites. After 3 years, given the absence of abdominal symptoms or abnormal blood test results, the patient underwent follow-up monitoring. However, given the patient’s young age, long-term follow-up is essential.

In conclusion, we present a case of grade III traumatic pancreatic injury involving main pancreatic duct damage, wherein initial attempts at transpapillarily bridging the injured pancreatic duct with the distal side using a pancreatic duct stent were unsuccessful. However, we succeeded in treating her with only NOM using alternative drainage methods, thereby obviating the need for surgery.
